# Advances of Genome Editing with CRISPR/Cas9 in Neurodegeneration: The Right Path towards Therapy

**DOI:** 10.3390/biomedicines11123333

**Published:** 2023-12-17

**Authors:** Aleksandr Klinkovskij, Mikhail Shepelev, Yuri Isaakyan, Denis Aniskin, Ilya Ulasov

**Affiliations:** 1 Group of Experimental Biotherapy and Diagnostics, Institute for Regenerative Medicine, World-Class Research Centre “Digital Biodesign and Personalized Healthcare”, I.M. Sechenov First Moscow State Medical University, Moscow 119991, Russia; becrame@gmail.com (A.K.); denaniskin@yandex.ru (D.A.); 2 Center for Precision Genome Editing and Genetic Technologies for Biomedicine, Institute of Gene Biology, Russian Academy of Sciences, 34/5 Vavilova Str., Moscow 119334, Russia; 3 Institute for Regenerative Medicine, Sechenov First Moscow State Medical University (Sechenov University), 8 Trubetskaya Str., Moscow 119991, Russia; yuri.isaakyan@gmail.com

**Keywords:** neurodegenerative disorders, cellular senescence, neuroinflammation, genome editing, adeno-associated virus, lentivirus, CRISPR/Cas9

## Abstract

The rate of neurodegenerative disorders (NDDs) is rising rapidly as the world’s population ages. Conditions such as Alzheimer’s disease (AD), Parkinson’s disease (PD), and dementia are becoming more prevalent and are now the fourth leading cause of death, following heart disease, cancer, and stroke. Although modern diagnostic techniques for detecting NDDs are varied, scientists are continuously seeking new and improved methods to enable early and precise detection. In addition to that, the present treatment options are limited to symptomatic therapy, which is effective in reducing the progression of neurodegeneration but lacks the ability to target the root cause—progressive loss of neuronal functioning. As a result, medical researchers continue to explore new treatments for these conditions. Here, we present a comprehensive summary of the key features of NDDs and an overview of the underlying mechanisms of neuroimmune dysfunction. Additionally, we dive into the cutting-edge treatment options that gene therapy provides in the quest to treat these disorders.

## 1. Introduction

**Figure 1:**
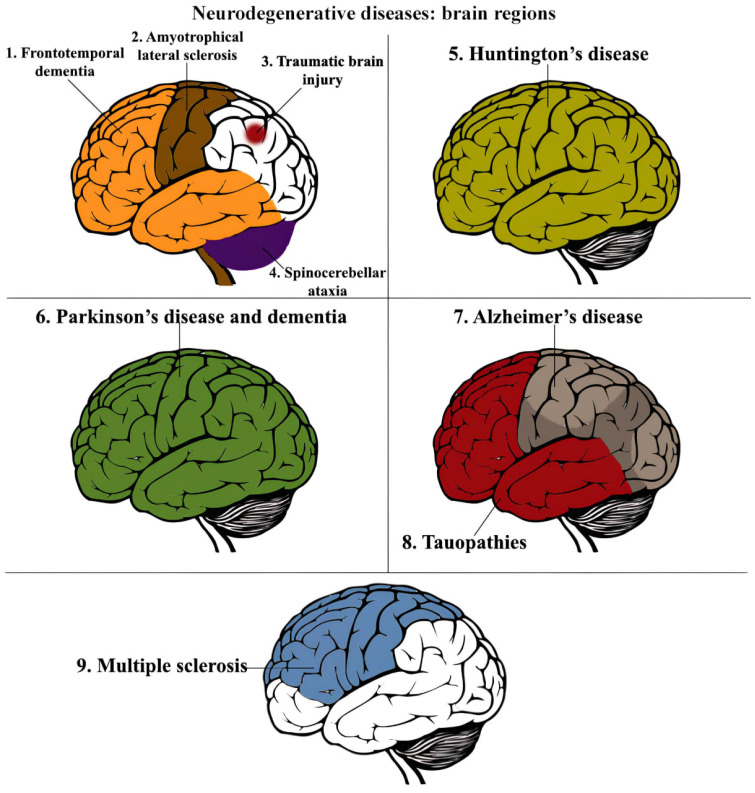
Association of gene disfunction, brain anatomic region localization, and type of brain neurodegenerative disorder. 1. *Genetics*: GRN, MAPT (tau), CSORFT2, VCP, CHMP28, SOSTM1, UBQUN2, TBK1. *Aggregating proteins*: TDP43, tau, SOD1, FUS, DPRs; 2. *Genetics*. ALS: SOO (SOD1), FUS, TARDP (TDP43), CSORFT2, UBCKN2, VCP, TBK1, ANXA11, PFN1, KFSA, VAPB, HNRNPA1, SOSTM1, NEK1, OPTIN, TUBAAA. *Aggregating proteins*: TDP43, tau, SOD1, FUS, DPRs; 3. *Aggregating proteins:* tau, Aβ; 4. *Genetics*: SCA1-3, SCA 7 (Ataxin1-3,7). *Aggregating proteins*: Ataxin, PolyQ; 5. *Genetics*: HTT (Htt). *Aggregating proteins:* Huntingtin (Htt), PolyQ; 6. *Genetics:* SNCA (a-syn), PINK1, PARK7/DJ1, PRKN/Parkin, ATP13A2, VPS35, LRRK2, GBA. *Aggregating proteins:* a-synuclein (dementia); 7. *Genetics*: APP (Aβ), PSEN1, PSEN2. *Aggregating proteins:* tau, Aβ; 8. *Genetics:* MAPT (tau). *Aggregating proteins:* tau [2]. 9. *Genetics:* HLA-DRB1, IL-7R. *Aggregating proteins:* PRIOC10, IgG1, IgG3, CSF Aβ.

As the global populace is growing older, there is a rapid increase in the incidence of neurodegenerative disorders (NDDs), such as Alzheimer’s disease (AD) and Parkinson’s disease (PD). It is worth mentioning that in developed countries, the number of dementia cases is projected to increase from 13.5 million in 2000 to 21.2 million in 2025 and 36.7 million in 2050 [1]. NDDs have a negative impact on various cognitive functions, such as memory, perception, coordination, fine motor skills, and thinking. Consequently, they lead to both short-term and long-term disorders and disabilities. Right now, these conditions rank as the fourth main cause of mortality, following heart disease, stroke, and cancer. This category encompasses debilitating conditions like AD, PD, dementia, and multiple sclerosis (MS), as well as other rare disorders such as amyotrophic lateral sclerosis (ALS), Huntington’s disease, and prion disorders. The neuropathologic assessments of these disorders are documented in Figure 1 [2].

Modern diagnostic methods for neurodegenerative disorders include the analysis of blood and cerebrospinal fluid for various biomarkers [3,4,5], neuroimaging using magnetic resonance imaging [6] and positron emission tomography [7], and psychological and neuropsychological testing [8]. However, there is a constant search for new and improved diagnostic methods that can help with the early detection and accurate diagnosis of these disorders. Currently, researchers are exploring the use of machine learning methods [9,10], immunohistochemistry, and new biomarkers in the blood associated with NDDs [11]. These new methods could potentially provide a more accurate and reliable diagnosis, as well as help monitor disease progression and treatment response. While diagnosis is of great interest, it is also important to consider treatment options for NDDs. Currently, treatment options are limited to symptomatic therapies such as pharmacological treatments [12,13] and cognitive and physical rehabilitation procedures [14,15]. Despite their effectiveness in slowing neurodegeneration, these methods do not address the underlying cause, which is the gradual loss of neurons. Therefore, the search for new treatment methods is crucial. The advent of gene therapy and other innovative treatments hold promise for enhancing patient outcomes and quality of life. Nonetheless, it is crucial to acquire a comprehensive understanding of the fundamental mechanisms underlying NDDs and recognize their distinguishing characteristics before embarking on the development of new therapeutic interventions. By targeting the hallmarks of NDDs, it may be possible to develop therapies that address the root cause of the disease, rather than just its symptoms.

## 2. Hallmarks of Neurodegenerative Disorders

Despite differences in the regions affected, NDDs exhibit notable resemblance in etiology at both the cellular and molecular levels. The following hallmarks have been identified:

- Damage to RNA and DNA [16,17];
- Disrupted proteostasis [18];
- Metabolic changes in neuroimmune cells that result in morphological alterations in glial cells and the microenvironment of the neuroimmune system [19];
- Oxidative and endoplasmic reticulum stress [20,21];
- Chronic inflammation, which was traditionally viewed as a protective function of the body but is now recognized as a hallmark of NDDs. Chronic inflammation can lead to focal cell death as a containment strategy, limiting the ability of pathogens and oncogenic cells to divide and spread. And this can manifest into NDDs [22].

Various intracellular and extracellular factors can induce cellular senescence, which is a mechanism associated with many aspects of NDDs. Senescence is characterized by an irreversible halt in cell cycle [22] and the emergence of a unique secretory phenotype [23]. Recent studies targeting senescent cells expressing the inhibitory protein p16^Ink4a^ have shown that the clearance of these cells delays the development of NDDs in mouse models [24,25].

These discoveries hold great importance as they provide insight into the inflammation hypothesis and introduce a fresh perspective on the significance of inflammation in the context of NDDs. During cellular aging, the continuous process of inflammation in senescent microglial and astrocyte cells leads to a constant release of pro-inflammatory cytokines, a process called inflammaging [26]. Subsequent to the presence of risk factors, the compensatory mechanisms of senescent cells become unregulated, initiating a cycle that actively contributes to the progression of NDDs [27].

## 3. Neuroimmune Dysfunction

The neuroimmune system is made up of three key components:

1. Microglia, which play an active role in the immune response of the central nervous system by producing pro-inflammatory and anti-inflammatory cytokines (M1 and M2 subtypes of microglia) [28].
2. Astrocytes, which regulate the restoration of the nervous system through their control over biochemical processes in epithelial cells of the blood–brain barrier (BBB) and their activation of the repair and scarring processes following the innate immune response [29].
3. Oligodendrocytes, which provide support, protection, and growth of axons [30].

Recognizing that the neuroimmune system functions as an intricately interconnected network of cells and signaling pathways crucial for maintaining nervous system homeostasis, it becomes evident that impairments within these components significantly contribute to the emergence of NDDs [28,31,32]. This emphasizes the need to comprehend and tackle the fundamental mechanisms involved in these disorders.

### 3.1. Effect of Microglia in Neuroinflammation

Neuroinflammation is a prominent characteristic observed in diverse NDDs. It involves the activation of microglia and astroglia cells, leading to the release of pro-inflammatory mediators such as reactive oxygen species, nitric oxide, TNF-α, IL-1β, and IL-6 [33]. The initiation of neuroinflammation can be stimulated by Pathogen and Damage-Associated Molecular Patterns (PAMPs and DAMPs) [34], with DAMPs stimuli being associated with misfolded and aggregated proteins [35]. These stimuli trigger a response in cells within the central nervous system (CNS) that primarily express pattern recognition receptors (PRRs) like microglia and, to a lesser extent, perivascular and peripheral macrophages, as well as other glial cells and neurons [33,36,37]. PRRs assume various forms of receptors, forming receptor complexes, which encompass scavenger and Toll-like receptors (TLRs). These receptor complexes initiate a signaling cascade that stimulates inflammation, triggering microglial activation and recruitment. As a result, neurotoxic molecules are generated, initiating the process of neurodegeneration [38,39,40,41]. The continuous presence of DAMPs in NDDs causes ongoing neuroinflammatory responses. This interaction between DAMPs and neuroinflammation leads to changes in microglia. The release of pro-inflammatory cytokines leads to a vicious cycle, causing harmful effects on brain function and overall health which ultimately lead to neurodegeneration.

Recent research has unveiled the presence of a specific group of microglia called disease-associated microglia (DAM). These microglia exhibit distinct patterns in gene expression and functionality, particularly that occur in NDDs [42,43]. The transformation of microglia into DAM begins when receptors on microglia identify specific molecular patterns associated with neurodegeneration (NAMPs), prompting their transition into the DAM state [42,43]. The DAM phenotype is observed in various NDDs such as AD, PD, and ALS. And more importantly, it is also detected in aged microglia [43,44].

### 3.2. Astroglial Scar Formation

Astroglial scar formation is a natural response to injury in the CNS. When an injury happens, astrocytes become active and start to proliferate. They form a protective scar barrier, which helps to prevent additional harm to the nearby cells [45]. However, in disorders, such as AD and PD, the formation of these astroglial scars can actually exacerbate the patients’ condition rather than providing a beneficial effect [46].

Neurodegeneration recruits inflammation-inducing immune cells as well as stromal fibroblasts and astrocytes that produce extracellular matrix (ECM). After neurons are damaged, they cannot be replaced by new ones. Instead, scar tissue forms and remains in place of the damaged neurons [46,47].

Fibrotic scarring in the CNS can arise as a result of diverse factors, including infection, parasites, and injury. When it comes to acute brain and spinal cord injuries, a particular type of fibrotic scar is formed, characterized by the presence of essential elements such as the ECM, myofibroblasts, and astrocytes. These components contribute to the structural integrity of the scar tissue in the affected area. However, in the context of chronic NDDs, the formation of a fibrotic environment is not as discernible compared to acute injuries. In these disorders, the scarring process and its components may differ, and the fibrotic environment may manifest in a more subtle manner.

Research suggests that with continued neuron loss, the damaged tissue is gradually replaced by elements of the ECM that are generated by various activated cells such as astroglia and fibroblasts [46]. This can result in a secondary immune response characterized by activation of microglia and peripheral immune cells. Although there are resemblances in the responsive cells and mediator reactions seen in both acute injuries and chronic disorders like ALS, MS and AD, it is essential to acknowledge that the severity and extent of fibrotic scarring can differ greatly.

Overall, the role of astroglia scar formation in neurodegenerative disorders is complex and requires further research to fully understand its impact on disease progression. Developing strategies to modulate astrocyte activation and scar formation through gene therapy may provide new avenues for treating these disorders [48].

### 3.3. Oligodendroglia and Myelin

The myelination process of axons in the nervous system plays a vital role in facilitating rapid impulse propagation. In terms of development, the peripheral nervous system (PNS) undergoes initial maturation, followed by the spinal cord and brain. Certain types of glial cells, namely oligodendrocytes in the CNS and Schwann cells in the PNS, have a crucial function in the process of axon ensheathment. They conduct a circular wrapping around a segment of an axon multiple times, creating multiple layers of myelin sheath using their plasma membranes. These complexes between axons and oligodendrocytes are referred to as “internodes” [49]. Most of the longitudinal extension of internodes coincides with secondary axon elongation during body growth, which lasts decades in humans [50]. In the human neocortex, myelination continues at least until the end of the second decade [51].

In a normal CNS, oligodendrocytes act as trophic support to axons by supplying them with energy. They do so by using monocarboxylate transporters (MCTs) to transfer energy-rich molecules (e.g., lactate) from the oligodendroglial compartment to the axonal compartment [52]. Lactate is transported into the space between the internode and axon, called periaxonal space, through the MCT1. Once inside the space, it is then taken up by the MCT2 and utilized as a fuel source for mitochondrial respiration, which helps in maintaining the energy demands of the axon. Apart from the metabolic aspect, myelin basic protein (MBP) is an essential component for the formation of compact myelin, which surrounds and insulates axons in the CNS. MBP acts as a “zipper” by helping in the compaction of the multiple layers of myelin membrane.

In NDDs such as AD, ALS, and multiple system atrophy (MSA), pathologic proteins accumulate in the periaxonal space and oligodendroglial cytoplasm, inhibiting motor transport and the free diffusion of lactate to axons [53]. In conducted research, it has been shown that a decrease in the expression of MCT1 in oligodendrocytes would diminish their capacity to provide lactate to axons and thereby contribute to the development of ALS [54]. Demyelination is observed in all three disorders and is associated with an autoimmune reaction to MBP in ALS and MSA [55]. The immune response then leads to an autoimmune reaction against oligodendrocyte precursor cells (OPCs) [56], further reducing the ability of oligodendrocytes to support axons. Impaired metabolic supplementation, an autoimmune reaction, and mitochondrial dysfunction lead to a decline in ATP concentrations and subsequent axonal impairment, ultimately resulting in the development of neurodegenerative disorders. Molecules and cellular components supporting ALS, MS, and AD development are summarized in a Table 1.

**Table 1:** A 3D network of molecules and cellular components supporting ALS, MS, and AD development. The table summarizes the main cellular components, mediators, and ECM molecules involved in ALS, MS, and AD, as well as in acute CNS damage. (IL—Interleukin, MMP—Matrix metalloproteinase, PDGFRβ—Platelet-derived growth factor receptor beta, TGFβ—Tissue growth factor beta, GFAP—Glial fibrillary acidic protein, CXCL—CXC chemokine ligand, NGF—nerve growth factor, TNFα—tumor necrosis factor alpha, PGD—prostaglandin, ADAMTS—Adamalysin-like metalloproteinases with thrombospondin motifs, CTGF—Connective tissue growth factor, CSPG—Chondroitin sulfate proteoglycan, HSPG—heparan sulfate proteoglycan. To explore a wider range of specific molecules that control or impact CNS cellular reactions to acute response, refer to a comprehensive analysis of the subject [57].

**CNS disease**			
Acute damage	Amyotrophic lateral sclerosis	Multiple sclerosis	Alzheimer’s disease
**Responder cells**			
Astrocytes, microglia, meningeal cells, fibroblasts	Astrocytes, microglia, meningeal cells, fibroblasts, oligodendrocytes	Astrocytes, microglia, meningeal cells, fibroblasts, oligodendrocytes, endothelial cells	Astrocytes, microglia, fibroblasts, smooth muscle cells
**Cell mediators and biomarkers**			
Thrombin, MMP-9, ATP, PDGFRβ, TGFβ, *GFAP*	IL-6, CXCL1, CXCL10, CXCL12, TNFα, TGFβ, NGF, INFγ, PGD2, ADAMTS-4, CTGF, S100A4, MMP-9, *GFAP*	PDGFRβ, TGFβ, myelin, *GFAP*	PDGFRβ, TGFβ, *GFAP*
**Extracellular matrix proteins**			
Fibronectin, laminin, collagen, CSPGs, tenasein, HSPGs	Fibronectin, collagen type IV, CSPGs, Sema3A, fibrin, vimentin, thrombin	Fibronectin, collagen, biglycan, decorin, CSPGs	Fibronectin, collagen, biglycan, decorin, CSPGs

## 4. New Types of Treatment: Gene Therapy and Genome Editing Technologies

Viral vector gene transfer refers to the use of viruses as vectors to deliver therapeutic genes or gene-targeting tools to specific cells within the body. This method holds great potential for the treatment of neurological disorders [58]. This can be primarily attributed to the factors listed below:

- Viral vectors can efficiently deliver therapeutic cargo to target cells and ensure its sustained presence over an extended period. This is important for diseases requiring long-term treatment, such as chronic neurological disorders;
- Viral vectors’ ability to efficiently infect postmitotic cells, including neurons in the brain, is a valuable characteristic. Many neurological disorders involve dysfunctional or damaged neurons, and viral vectors offer an effective means of delivering therapeutic cargo directly to these cells;
- Viral vectors used in gene transfer have been engineered to have low immunogenicity, meaning they are less likely to trigger an immune response. Additionally, extensive research has focused on reducing the toxicity associated with viral vectors, making them safer for use in gene therapy;
- Viral vectors’ compatibility with other forms of therapy approaches, including pharmacological treatments or surgical interventions. This compatibility allows for combination therapies that may enhance overall treatment outcomes.

By understanding the biology of these viral vectors, researchers and healthcare professionals can harness their advantages to develop therapeutic interventions for various neurological disorders. It is important to note that while viral-mediated gene transfer holds immense promise, ongoing research aims to optimize its safety, efficiency, and specificity to ensure its successful application in medicine.

### 4.1. Lentiviral Vectors

Studies have demonstrated that lentivirus (LV)-based vectors can effectively transduce various types of brain cells, including neural stem cells, neurons, astrocytes, and oligodendrocytes [59,60,61,62]. Moreover, these vectors have the capability to sustain long-lasting expression of the transgene within the brain [63,64]. This is particularly significant for gene therapy applications in the CNS because it allows for extended production of the desired therapeutic gene following a single administration of the virus.

LVs are derived from HIV-1 and have been extensively modified to remove all open reading frames (ORFs) of HIV-1, making them safe and suitable for use in gene therapy and other research applications [65]. The genome of lentiviral vectors is composed of approximately 10.7 kilobases of positive-sense single-stranded RNA (+ssRNA). This RNA carries the genetic information that needs to be delivered to the target cells and can be immediately translated by the host cell (therefore called the positive-sense). The viral genome is enclosed in a spherical lipid-enriched viral envelope, which gives the LVs a diameter of about 100 nanometers. Although the ORFs of HIV-1 have been removed, they retain several crucial non-coding elements, allowing them to accommodate genetic material of up to 10 kb [58]. The lentiviral genome contains regions responsible for structural, enzymatic and enveloping functions such as group-specific antigen (gag), DNA polymerase (pol), and envelope (env). The gag genes encode three structural proteins: viral matrix, capsid, and nucleoproteins. These proteins are essential for the assembly and structural integrity of the viral particle. The pol gene encodes the enzymatic function of the virus, including reverse transcriptase (conversion of the viral RNA into DNA), protease (processing of viral polyproteins, facilitating the maturation of the virus), and integrase (integrating the viral DNA into the host cell genome, enabling long-term expression of the delivered genetic material) [58]. In addition to the genetic components, lentiviral vectors utilize their viral envelope (env) for attachment to and entry into the host cells. The env protein, derived from the HIV-1 envelope glycoprotein, enables the vector to recognize specific receptors on the surface of the target cells. This interaction triggers the internalization of the vector into the host cell, allowing for the delivery of the genetic payload.

One of key steps in expanding the tropism of LVs, or the ability to infect different cell types of lentiviral vectors, involves pseudotyping viral particles with heterologous envelope proteins. This technique has significantly improved the safety profile of the vectors and expanded the range of target cells that can be transduced [66]. Pseudotyping refers to the process of replacing the native envelope protein of the lentiviral vector with a different envelope protein derived from another virus. This modification allows the lentiviral vector to interact with specific receptors on the surface of target cells recognized by the newly introduced envelope protein. This technique has significantly improved the safety profile of the vectors and expanded the range of target cells that can be transduced [67]. The pseudotyping strategy has enabled lentiviral vectors to be pseudotyped with a wide variety of envelope proteins, broadening the tropism of these vectors and making them more versatile for different applications. Different envelope proteins can confer specific tropism to the lentiviral vector, allowing for targeted gene delivery to a particular cell type or tissue. Many of these envelope proteins, such as vesicular stomatitis virus G-protein, lymphocytic choriomeningitis virus, Mokola virus, Ross River virus, and Rabies virus, have shown strong neurotropic tropism [68].

One of the challenges with the clinical use of LVs is the potential risk of insertional mutagenesis [69]. Insertional mutagenesis can occur when the vector integrates into the host cell genome, potentially disrupting normal gene function or triggering unintended consequences. To address this concern, integrase-deficient lentiviral vectors (IDLVs) have been developed [70]. These vectors contain mutations in the integrase enzyme, which is responsible for integrating the viral DNA into the host cell genome. The integrase mutations prevent proviral integration, resulting in the production of elevated levels of circular vector episomes in the transduced cells. Vector episomes are small circles of DNA that do not integrate into the host cell genome. They are able to persist independently in the transduced cells and can maintain stable transgene expression. Importantly, IDLVs lack replication signals, which means they are unable to replicate and spread within the host cell. This characteristic enhances their safety profile. However, while IDLVs are quite stable in quiescent cells, their episomes are gradually diluted in dividing cells, leading to their loss over time [71]. The ability to produce episomes and their eventual loss in dividing cells make IDLVs favorable for certain applications, including NDDs. In tissues where long-term gene expression is not required or where transduced cells are expected to be quiescent (e.g., certain regions of the nervous system), lentiviral vectors can provide efficient, controlled gene delivery with reduced risk of insertional mutagenesis.

### 4.2. AAV Vectors

Adeno-associated viral (AAV) vectors have become the most commonly used platform for therapeutic gene delivery due to their safety and efficiency [72]. These vectors were developed from a wild-type virus that was originally discovered in 1965 and belongs to the genus *Dependoviruses* of the family *Parvoviridae* [73].

One of the unique features of AAV is its requirement for coinfection with another virus, such as adenovirus or herpes simplex virus, in order to replicate in host cells [74]. This dependence on a helper virus is one of the reasons why AAV is considered safe for gene therapy applications, as it cannot cause a productive infection without the presence of the helper virus. The AAV genome consists of a single-stranded DNA molecule that is approximately 4.7 kilobases (kb) in size [75]. The genome itself is relatively simple and contains two ORFs known as rep (replication and regulation) and cap (capsid proteins), flanked by pair of 145 base pair inverted terminal repeats, which are essential for the replication and packaging of AAV [76].

There are several reasons why AAV is considered an ideal virus as a delivery vector:

First is safety and immune response: AAV has a remarkable safety profile as it does not cause any significant pathologies in humans. When administered at acceptable dosages, it typically elicits only a mild immune response. This is an important characteristic since a strong immune response can hinder transgene expression and potentially lead to adverse effects [77].

Second is long-term transgene expression: AAV genomes can be maintained in episomal forms within host cells for extended periods. This stability allows for sustained transgene expression, enabling long-term therapeutic effects. This is significant in gene therapy applications where persistent transgene expression is desired to achieve lasting therapeutic benefits [75].

Third is diverse serotypes and tropism: AAVs are naturally occurring viruses that are widespread in nature. Numerous AAV serotypes have been identified, each displaying distinct tissue tropism and preferred target cells. This broad range of serotypes allows for targeted gene delivery to specific tissues or cell types of interest, enhancing the precision and efficacy of gene therapy approaches [78].

Finally, the genome of AAV is well understood: The AAV genome is extensively studied and thoroughly understood. This knowledge facilitates the development of precise and predictable genetic manipulations, including modifications to the viral capsid for improved tissue targeting or evasion of the host immune response. The predictable outcomes of these manipulations enhance the safety and efficacy of AAV-based gene delivery [79].

According to recent research, the following rAAV type 2 vector serotypes have been shown to be effective in transducing cells of the CNS: AAV2/1, AAV2/5, AAV2/6, AAV2/8, and AAV2/9 [80]. Among these serotypes, AAV2/1 and AAV2/5 have been found to be more efficient than AAV2/2 at transducing neurons and glial cells in multiple brain regions of rats and nonhuman primates [81]. This suggests that AAV2/1 and AAV2/5 might be better candidates for targeting neuronal and glial cells in the CNS. On the other hand, AAV2/7, AAV2/8, and AAV2/9 seem to primarily transduce neuronal cells, with AAV2/9 exhibiting the widest spread from the injection site [82]. This indicates that AAV2/9 might be suitable for targeting neuronal cells and achieving broader distribution within the CNS. The variability in axonal transport among AAV serotypes provides an opportunity to infect not only the directly targeted cell types but also their projection fields. This means that by utilizing specific AAV serotypes, researchers can potentially target specific projection pathways and study circuitry within the CNS.

Identifying vectors capable of crossing the BBB is a challenge in targeting the brain for gene therapy. Intravenously administered AAV2/9 has been shown to cross the BBB in mice and cats, including neonatal and adult animals [83], while AAV2/8 achieves this to a lesser extent than AAV2/9 [84]. Importantly, intravenously injected AAV2/9 vectors transduce both neurons and astrocytes, demonstrating that gene therapy delivery to a significant extent of the brain and spinal cord is feasible without direct CNS injection [85].

AAV-based treatments for CNS disorders are indeed advancing and becoming a part of clinical practice. Luxturna, which is an AAV2-based gene replacement therapy, has been successfully used in the treatment of Leber congenital amaurosis. Luxturna delivers a functional copy of the RPE65 gene to the retinal cells, restoring their ability to produce the missing enzyme and improving vision [86]. Likewise, Zolgensma, an AAV9-based gene replacement therapy, has been used for the treatment of spinal muscular atrophy [87]. Zolgensma delivers a functional copy of the SMN1 gene to the target cells, effectively replacing the defective gene and improving motor neuron function. Furthermore, ongoing clinical trials are investigating the effectiveness of other AAV-based medications for various neurological conditions [88]. Clinical trials play a crucial role in testing the safety and efficacy of these therapies before they can be widely available for patients.

Despite their efficiency and good safety profile, the use of AAVs for gene replacement therapy or genome editing has two important drawbacks. First, AAVs are able to integrate into human genomes in the *AAVS1* locus, although with quite low frequency of around 0.1% [89]. Strikingly, a high level of AAV integration (up to 47%) into Cas9-induced double-strand breaks both in cultured neurons and in mice in in vivo was recently discovered upon the editing of therapeutically relevant genes such as *APP* [90]. These findings indicate that the outcomes of AAV-based gene therapeutic approaches should be carefully evaluated. Second, AAVs have only about a 4–4.4 kb packaging capacity that limits the capabilities of the simultaneous packaging of expression cassettes of genome editing nucleases, sgRNAs, and templates for DSB repair (see below), which should be provided as separate AAVs [91]. Noteworthy, several novel small nucleases (Un1Cas12f1, AsCas12f1, and others) have been recently discovered [92,93] and subsequently harnessed for efficient genome editing in human cells when delivered as AAVs [94,95]. These findings open new avenues for the development of efficient genome editing tools to treat NDDs.

### 4.3. Adenoviral Vectors

Adenoviral vectors are widely used viruses for gene delivery. Studies conducted in the late 1990s have shed light on the neural tropism of adenoviral vectors through both in vitro and in vivo experiments [96,97]. Adenoviral vectors are nonenveloped, double-stranded DNA viral vectors with a packaging capacity of approximately 35 kb. There are over 50 different serotypes of adenoviruses that are grouped into six species [98]. Among these serotypes, types 2 and 5 are frequently utilized to develop recombinant adenoviral vectors due to their low association with human ailments [99]. To maximize the packaging space in adenoviral vectors, the majority of them are engineered to be E1 gene-deleted, rendering the virus replication-deficient. Additionally, deletion of the E3 region is often performed to increase the carrying capacity. Adenovirus utilizes the cellular coxsackie and adenovirus receptor for attachment to the target cells and subsequently employs αv integrins for internalization. This feature presents a notable advantage of adenoviruses as it allows for a broad tissue tropism and increased expression profile, which contributes to their effectiveness in gene delivery combined with their packaging ability. Adenoviral vectors have several advantages, including their ability to hold large genes, high titers in production, and ease of purification. The HEK293 cell line, which stably expresses E1A and E1B genes, is commonly used for the replication and packaging of adenoviral vectors. One strength of adenoviral vectors is their robust transgene expression, which is particularly notable in first-generation vectors. However, it is important to note that transgene expression from these vectors tends to decline and cease around 2–3 weeks after direct injection. This limited duration of gene expression can be a significant challenge for certain applications where long-term expression is desired. The immunogenic nature of adenoviruses poses another hurdle for long-term gene expression. Adenoviral vectors can be recognized by the immune system, leading to immunological responses that may impair sustained transgene expression. This immune response can result in the clearance of vector-transduced cells and the generation of neutralizing antibodies against the vector [100]. Inflammatory cytokines, which are produced in a response to adenoviral infection, can also contribute to the termination of gene expression by affecting promoter regulation [101]. These cytokines can impact the activity of promoters driving transgene expression, further limiting the persistence of gene expression. Although adenoviral vectors can be useful research tools in vitro and in animal models as they are relatively straightforward to produce and offer high levels of transgene expression, mounting evidence suggests that their utilization in the nervous system comes with obstacles that pose a challenge for clinical translation [102]. Multiple attempts have been made to utilize adenoviruses in the treatment of NDDs, as discussed in the publication [103]. However, current research suggests that this approach may be limited by the immune system’s capacity to eliminate adenoviral transgene expression in the brain, as reported in the study of Lowenstein et al. [102]. Additionally, directly injecting viral particles into the brain can lead to an enhanced accessibility of the virus as well as immune stimulation, further discouraging the use of adenovirus-based gene therapy for treating NDDs.

### 4.4. Genome Editing Technologies

Zinc finger nucleases (ZFN) were the first programmable nucleases used in genome editing. ZNFs are composed of three zinc finger domains that specifically bind DNA sequences and the catalytic domain of FokI restriction endonuclease from *Flavobacterium okeanokoites* bacteria. FokI cuts DNA only as a dimer; therefore, for genome editing it is required to design two ZFNs recognizing targets on opposite strands and oriented in a convergent direction [104]. This would allow FokI domains to dimerize and make a double-strand break (DSB) in the targeted region. ZFNs have been used for genome editing in a variety of organisms, including mammals [105].

Another class of genome editing tools is TALENs, that represent a fusion between the TAL effector DNA binding domain from *Xanthomonas* bacteria and the catalytic domain of FokI. The DNA binding domain contains a repeated highly conserved 33–35 amino acid sequence with variable amino acids at the 12 and 13 positions called repeat-variable di-residues (RVDs) [106]. These two amino acids are subject to design and provide the DNA binding specificity. One repeat binds one nucleotide; hence, the repeats could be linked in a linear fashion to recognize almost any DNA sequence and induce double-strand break in this region via FokI [107]. Further modifications of the TALEN systems include the usage of other restriction enzymes such as PvuII, I-Ani, and some others, as well as the development of TALEN-base editors, transcriptional activators and repressors, and other applications [108].

Although ZNFs and TALENs allowed for targeted genome editing in mammalian cells with previously unavailable ease and specificity, these two systems were outperformed by the CRISPR/Cas9 system due to its high efficiency and unapparelled ease of design and use.

The field of gene therapy was revolutionized with the adaptation of the CRISPR/Cas system (Clustered Regularly Interspaced Short Palindromic Repeats/CRISPR-associated proteins) for genome editing in human cells [109,110]. The CRISPR/Cas system is an acquired-immunity mechanism in bacteria and archea that is used to eliminate the invading nucleic acids [111]. The system encompasses multiple components that work in diverse ways and is classified into two classes composed of six types (I–VI) of Cas proteins, with at least 29 subtypes [112]. Currently, class II type II *Streptococcus pyogenes* Cas9 (SpCas9) and class II type V *Acidaminococcus* sp. (AsCas12) are the most widely used Cas nucleases for both experimental and therapeutic purposes. Moreover, many other Cas proteins were harnessed for genome editing or RNA editing in mammalian cells. For example, recently discovered class V-F compact Cas12f nucleases hold promise for therapeutic usage due to their small size, allowing for efficient packaging into AAVs (see above) [92,93]. SpCas9 is an RNA-guided nuclease, which is targeted to a specific genomic locus via complementary binding of Cas9-bound short RNA to the 20-nt-long protospacer genomic sequence followed by the 3-nt-long NGG protospacer adjacent motif (PAM). In bacteria, the Cas9 nuclease forms a ribonucleoprotein (RNP) complex with two short RNAs: tracrRNA and crisprRNA (crRNA), where tracrRNA acts as a scaffold that binds Cas9 and crRNA pairs with tracrRNA and recognizes its genomic target. Subsequently, it was shown that tracrRNA and crRNA could be fused into a single guide RNA (sgRNA) [113], which greatly simplified the use of the CRISPR/Cas9 system in mammalian cells [109,110], although tracrRNA/crRNA-containing Cas9 RNPs are also widely used. Unlike SpCas9, AsCas12 and other Cas12 family nucleases do not require tracrRNA for processing CRISPR arrays; therefore, in mammalian cells Cas12 nucleases require only crRNA for efficient genome editing [114]. Mechanistically, to perform genome editing, the 20-nt-long part of sgRNA through base–base complementary pairing targets Cas9 to a specific genomic site, where Cas9 introduces DSB 3 nt upstream of the PAM. Next, the DSB is repaired by cellular DNA repair machinery via either non-homologous end joining (NHEJ) or homologous recombination (HR) pathways [115]. Error-prone repair via NHEJ leads to a formation of small insertions or deletions (indels) at the targeted genomic site, thus producing frameshifts and gene knock-out. If the exogenous DNA repair template is provided simultaneously with the CRISPR/Cas9 components, then it could be used by cellular DNA repair machinery as an instruction to introduce a template-encoded DNA sequence into the genome [116].

The ease of use and high efficiency of CRISPR/Cas systems stimulated the development of therapeutic applications of genome editing tools. To briefly summarize, the CRISPR/Cas9 system could be used for the inactivation of a mutated allele of the gene (e.g., disruption of the mutated *APP* allele to treat AD [117], for precise correction of point mutations (e.g., in patients with AD or PD [118]), for introduction of large DNA fragments into a predetermined genomic site (e.g., knock-in into *TRAC* locus for the development of CAR T-cell therapies [119]), for correction of alleles associated with MS [120], and many others).

To further broaden the applications of the CRISPR/Cas system, base editors, prime editors, gene regulation systems, and other genome editing tools were developed [116,121]. All these tools in the nearest future will likely find applications for NDDs treatment. Multiple reagent formats and delivery options exist to introduce therapeutic CRISPR/Cas reagents into target cells [122]. Cas9 and sgRNA could be delivered as an expression plasmid DNA or as Cas9 mRNA and sgRNA, or as a complex of Cas9 protein and sgRNA (RNP). The most attractive modes of CRISPR/Cas delivery from the therapeutic point of view are AAVs, LVs, Virus-like particles (VLPs), and RNPs; although, many other options exist [122]. The options of choice will depend on the mode of therapeutic intervention (ex vivo vs. in vivo), on the desired editing outcome (gene knock-out vs. knock-in), and so on.

Despite current progress in the development of CRISPR/Cas-based therapeutic tools, there are several obstacles on the way to efficient therapeutic genome editing. First of all, it is the issue of off-target genome modification that can lead to unwanted changes in the genomes, from point mutations to the loss of whole chromosomes [123]. To overcome these problems, high-fidelity versions of Cas9 and other nucleases with reduced off-target activity were developed [124,125,126]. Another important challenge is the efficient delivery of CRISPR/Cas9 nucleases into target human cells, especially for in vivo genome editing as well as an issue of tissue or cell-specific delivery.

Taken together, CRISPR/Cas genome editing tools when combined with efficient delivery systems will be allowed for the development of therapeutic tools to treat NDDs and efficiently create cellular and animal models for research purposes.

## 5. Neurodegenerative Disorders’ Therapeutic Targets and Their Application

To date, several potential markers have been identified as targets for gene therapy and/or genome editing for the treatment of NDDs.

### 5.1. Sox9

The *Sox9* is a transcription factor that facilitates the growth of stem and progenitor cells in pluripotent, fetal, and adult tissues. Its regulation is controlled by several signal transduction pathways, such as Sonic Hedgehog, Notch, TGF-β, and Fgf9-mediated signaling [127]. In embryonic dorsal root ganglia (DRG) of sensory neurons, the expression of Sox9 is lowered [128]. However, it is expressed in astrocytes and ependymal cells within the neurogenic regions of adult human and mouse brains [129]. Sox9 plays a critical role in upregulating genes associated with glial scar formation. Glial scars, characterized by the accumulation of chondroitin sulfate proteoglycans (CSPGs), are known obstacles to axon regeneration in the CNS. A study has shown that inhibiting Sox9 expression in glial cells through tamoxifen-inducible Sox9 deletion in adult mice has been shown to have beneficial effects on scar formation and functional recovery after spinal cord injury [130]. This study demonstrated that when Sox9 expression was specifically inhibited in glial cells in adult mice, there was a resulting decrease in CSPG production and smaller scar sizes in the spinal cord following injury. This reduction in scar formation was accompanied by improved motor function recovery. These findings suggest that targeting Sox9 expression in glial cells could be a potential strategy for promoting functional recovery by mitigating the inhibitory effects of glial scars on axon regeneration.

### 5.2. RGMa

Repulsive guidance molecule A (RGMa) plays a role in axon guidance and recovery after spinal cord injury. RGMa is a glycosylphosphatidylinositol-linked, membrane-associated protein that binds to its receptor Neogenin (Neo1) to regulate axon guidance. After spinal cord injury, RGMa is not only upregulated in neurons but also in other cell types such as oligodendrocytes, astrocytes, activated microglia, and macrophages. This broader upregulation suggests that the effects of RGMa on axon regeneration and recovery after spinal cord injury involve multiple cell types and signaling pathways [131] and therefore suggests that it may create an inhibitory environment for axon regeneration in the injured spinal cord. In a study utilizing monoclonal antibodies as a targeted approach to counteract the inhibitory effects of RGMa, mice were able to potentially restore axonal connections and recover lost functions after 6 weeks of prior injury [132]. This suggests that targeting RGMa could be a potential therapeutic strategy for promoting axon regeneration and functional recovery. Furthermore, it is interesting to note that RGMa-Neo1 signaling has been shown to promote cell survival after optic nerve transection in adult rats. However, it does not appear to promote axon regrowth in this context. This suggests that the role of RGMa-Neo1 signaling might vary depending on the specific injury model and cellular context. The coregulation of RGMa and Neogenin also plays a significant role in neurogenesis [133,134], neural tube morphogenesis [127], and neural tube closure through the RhoA/ROCK pathway [127,132]. Further research investigating the role of the RGMa-BMP [133] pathway in neurogenesis and its therapeutic potential is necessary for better understanding of this area. Finally, RGMa is also found to be upregulated in NDDs such as MS and PD [128,135,136]. These findings present a promising therapeutic avenue for neutralizing anti-regenerative factors, thereby promoting axon re-extension after injury [131].

### 5.3. MAG

Myelin-associated glycoprotein (MAG) is a transmembrane glycoprotein that is primarily produced by myelinating glial cells, specifically oligodendrocytes in the CNS and Schwann cells in the PNS. Its main role is in the maintenance of myelinated axons. MAG was indeed the first myelin-associated inhibitor to be molecularly characterized [137]. MAG plays a crucial role in the maintenance of myelinated axons. It is localized in the innermost portion of the myelin sheath, where it interacts with the axon membrane. MAG’s function is thought to involve stabilizing the myelin sheath structure and promoting the adhesion between myelin and the axon. These interactions contribute to the integrity and proper functioning of myelinated axons in both the CNS and PNS [138]. One fascinating aspect of MAG’s effects on axon growth is its bimodal nature. It can have different effects on axon growth depending on the age and type of neurons involved. In particular, MAG has been found to promote axon growth in young neurons while inhibiting growth in older neurons. This age- and neuron-type-dependent switch in MAG’s effects on axon growth adds another layer of complexity to its function [137]. The bimodal effects of MAG have been experimentally validated using neurite growth assays with postnatal and adult neurons. In these assays, MAG is commonly used as an inhibitory substrate to study the effects of myelin-associated inhibitors on neurite outgrowth. It has been a valuable tool in understanding the inhibitory factors present in myelin and their impact on axonal regeneration. While MAG’s role in promoting or inhibiting axon growth has been extensively studied in vitro using neuron cultures and in development, there have been relatively few studies that have examined its function in axonal growth following injury in vivo. The available studies paint a complex picture of MAG’s role in the CNS. Several genetic studies have been conducted to investigate the effects of knocking down MAG on axon regeneration and have shown that MAG alone does not lead to improved axon regeneration [139,140,141]. This contradictory result indicates that MAG may have opposing functions even in the mature CNS, inhibiting the growth of some neurons while promoting the growth of others. This suggests that the effects of MAG on axon growth may be highly context-dependent, influenced by the specific neuronal populations involved and the injury or disease conditions. Beyond its effects on axon growth, MAG has also been implicated in mediating axon stability and integrity and protecting axons under pathological conditions. Studies have shown that MAG may have a protective role in the CNS by promoting axonal stability and preventing further degeneration [142,143]. Genetic deletion of MAG in animal models resulted in accelerated axonal loss in experimental autoimmune encephalomyelitis (model of MS), supporting the notion that MAG plays a role in protecting axons in disease conditions [144]. These findings suggest that MAG’s functions in the axonal response to injury and disease are more complex than initially recognized. In addition to its well-established role in growth inhibition, MAG may have additional roles in promoting axonal growth and protecting axons from further degeneration in the mature CNS [145].

### 5.4. Lin28

Lin28a, along with its paralog Lin28b in vertebrates, is a highly conserved RNA binding protein. It plays a crucial role in the development of the embryo and fetus and is mainly expressed during the early stages [146]. According to recent research [147], Lin28a is involved in the self-renewal of neural stem and precursor cells and in promoting the differentiation of neurons in the developing brain. In CNS tissues, where levels of Lin28 are typically low after injury [148], the use of an AAV to produce overexpression of Lin28 has been shown to improve long-distance axon regeneration in both corticospinal and retinal ganglion cell neurons [149,150]. This suggests that manipulating Lin28 activity in the aftermath of CNS damage could be a viable option for promoting tissue regeneration and treating injuries. However, it should be noted that, like mTOR, boosting levels of Lin28 in humans could also pose a risk of oncogenesis [151].

### 5.5. Notch1

Notch1 is a crucial factor in the maintenance of hematopoietic stem cells, preventing differentiation and promoting self-renewal capabilities [152,153]. It is also an effective marker utilized in the differentiation of cancerous and non-cancerous stem cells [154,155]. In the nervous system, Notch1 is essential for the differentiation of neural and glial cells, along with modulating activity-induced synaptic plasticity [156,157,158]. Recent studies have demonstrated how Notch1 signaling can negatively impact axon regeneration and inhibiting it can promote axon regeneration [159]. On the other hand, the injury-induced Notch activation mechanism and Notch intracellular domain target genes are yet to be explored. Various ligands help regulate the Notch pathway [160], and the identification of such injury-dependent Notch ligands is necessary. Additionally, in various spinal cord injury models in mice, Notch1 upregulation is seen exclusively in neurons [161,162,163], further emphasizing the need for additional research.

### 5.6. Msi1

Msi1 (Musashi RNA binding protein 1) is an RNA binding protein that plays a crucial role in regulating the translation of target mRNAs; it is also highly expressed in the CNS [164,165,166]. One of the key functions of Msi1 is its involvement in the proliferation of neural progenitor cells (including CNS stem cells). Neural progenitor cells have the ability to self-renew and differentiate into different cell types in the CNS. Msi1 plays a crucial role in maintaining the stemness of these cells and controlling their proliferation. Studies have demonstrated its instrumental role in maintaining the stemness of these cells and regulating their self-renewal and differentiation [167]. It does so by binding to specific target mRNAs and modulating their translation. By regulating the expression of these target mRNAs, Msi1 influences the fate decisions of neural progenitor cells, ensuring an appropriate balance of self-renewal and differentiation. Msi1 has also been found to bind to Robo3, contributing to the regulation of posttranscriptional events. This regulatory interaction is essential for the proper midline crossing of pre-cerebellar neurons [168].

In addition to its crucial roles in neural development and stem cell regulation, Msi1 has emerged as a significant marker of regeneration in amphibians. Levels of Msi1 expression in spinal cord ependymal cells have been found to differ between amphibians with defective regeneration and those with robust regenerative capabilities [169]. This finding highlights the potential involvement of Msi1 in axon regeneration and regenerative processes. Further evidence of the importance of Msi1 in axon regrowth comes from functional screening studies in nematodes. Msi1 was as an essential factor for axon regrowth in nematodes. This discovery reinforces the notion that Msi1 plays a conserved role in promoting axon regeneration across different species [170]. Based on these two statements, manipulating Msi1-dependent cellular stemness and its regulatory functions could be a potential target for promoting axon regeneration. By understanding the molecular mechanisms underlying mRNA stability, translational control, and localization associated with axon regeneration, researchers may be able to develop strategies to enhance the regenerative capacity of neurons.

### 5.7. Prom1

Prom1 is a membrane glycoprotein with a pentaspan structure which is known to have an affinity for cholesterol. It is utilized as a marker for adult stem cells or cancer stem cells [171,172,173] and is essential for the formation of membrane-protruding structures like tunneling nanotubes [174]. Deletion of the Prom1 gene results in neural defects, such as retinal degeneration [131], decline in the number of brain neurons, and walking difficulties [175,176,177]. Hence, Prom1 might play a significant role in neural tissues. Transplantation of peripheral blood prominin1-positive cells to mice has shown to promote neural regeneration, functional recovery, and neural integrity through angiogenesis, astrogliosis, and axon growth in injured spinal cords in mice [178,179,180,181]. Although Prom1′s expression is downregulated during DRG neurons’ development [128], Prom1 still exists during adult stages [132], playing a critical role in the differential regulation of genes linked to cholesterol metabolism [132]. Overexpression of Prom1 stimulates axon regeneration in vivo and in vitro and reducing cholesterol in DRG neurons enhances axonal regeneration in vitro, suggesting the cholesterol level to be a potential target to manage axonal growth [132]. Although, Prominin1′s role in gene expression regulation in DRG neurons remains unknown, requiring further exploration to fully grasp functions of Prom1 in neuronal and non-neuronal cells like cancer stem cells.

In addition to studying biomarkers individually, there is a rising fascination with deploying sophisticated techniques that merge stem cell biology with gene therapy, for instance, OSKM (OCT4, SOX2, KLF4, and MYC) transcription factor reprogramming [182] and microglia replacement therapy [183,184].

## 6. Overview of Clinical Trials for Treatment of NDDs

Gene therapy has advanced rapidly, opening up possibilities for understanding and treating NDDs. However, translating these advancements into effective clinical therapies may be challenging and not easily achievable in the near future. The field of gene therapy for NDDs has faced three major challenges. Firstly, delivering therapeutic genes to the targeted cells within the CNS has proven to be a significant hurdle due to barriers like the BBB [185]. Secondly, achieving persistent and long-term expression of introduced genes in the target cells has posed a challenge as sustained expression is crucial for achieving lasting therapeutic effects [186]. Lastly, managing immune responses and addressing safety concerns has been important as the immune system may recognize the gene therapy vectors as foreign, resulting in potential inflammation and harm. Overcoming these challenges has been crucial for advancing the field of gene therapy for NDDs.

Currently, there are 39 active clinical trials registered on clinicaltrials.gov focusing on gene therapy for NDDs. These trials explore different therapeutic approaches, including the delivery of AAVs and other gene therapeutics.

Genome editing technologies show promises for the treatment of cancers, blood disorders, viral infections (HIV), and some other diseases [187,188]. Currently there are 133 records of clinical trials using genome editing technologies; most of them are based on the CRISPR/Cas9 system, only 20 are based on the use of ZFNs, and 8 are based on TALENs [187]. These data clearly indicate that the CRISPR/Cas9 system currently is the method of choice for the development of genome editing therapeutics.

The CRISRP/Cas9 system has been widely used for pre-clinical studies and the development of therapeutic strategies to treat NDDs [189], but so far none of the putative genome editing drugs have reached clinical trials for the treatment of NDDs.

Further efforts are needed to overcome limitations imposed on gene therapy and genome editing approaches for treatment of NDDs. We believe that the future of genome editing for therapeutic purposes is optimistic, taking into account the recently approved CRISPR/Cas9-based therapeutic CASGEVY [190]. However, evaluating the clinical transformation of genome editing tools and further advancements are necessary. Collaborative research efforts and large-scale studies are crucial for advancing the field of genome editing for the treatment of NDDs.

## 7. Conclusions

The process of neuron regeneration in adults presents challenges due to molecules in the brain that inhibit the process and the limitations of current scar treatment therapy and stem cell availability. However, gene therapy offers promising potential to address these challenges. Clinical trials that aim to treat NDDs may have flawed results due to several factors, including insufficient data from prior animal studies, suboptimal cassette design of the expression vector, and immunogenicity of the delivery vector or therapeutic transgene [191]. Among the 9008 trials registered on the Clinicaltrials.gov database for treating NDDs worldwide, 186 involve gene therapy approaches, where 47 of them use viral delivery methods. Although delivery of genetically engineered medications directly to brain cells using LV and AAV vectors is being explored as a possible option, all recent gene therapy discoveries have their own limitations, including bioethics concerns which need to be addressed prior to testing in clinical settings [192]. Additionally, CRISPR/Cas9 technology has the potential to revolutionize direct genome editing in patients with NDDs. Moreover, gene therapy can be employed to alleviate symptoms in the initial stages by stimulating molecule production to reduce inflammation and enhance neuron performance. In conclusion, treatment of NDDs is an important area of scientific research, and gene therapy offers potential to address the challenges presented. Ongoing research into delivery methods and genome editing techniques will likely lead to new breakthroughs in this field.

## Data Availability

No new data were created or analyzed in this study. Data sharing is not applicable to this article.

## Abbreviations

AAV	adeno-associated virus
AD	Alzheimer’s disease
ALS	amyotrophic lateral sclerosis
BBB	blood brain barrier
CSPG	chondroitin sulfate proteoglycans
CNS	central nervous system
CRISPR/Cas	clustered regularly interspaced short palindromic repeats/CRISPR-associated protein
crRNA	crisprRNA
DAM	disease-associated microglia
DAMP	damage-associated molecular patterns
DRG	dorsal root ganglion
DSB	double-strand break
IDLV	integrase-deficient lentivirus
ECM	extracellular matrix
HR	homologous recombination
LV	lentivirus
MAG	myelin-associated glycoprotein
MBP	myelin basic protein
MCT	monocarboxylate transporter
MSA	multiple system atrophy
MS	multiple sclerosis
NAMP	neurodegeneration-associated molecular patterns
NDD	neurodegenerative disorder
NHEJ	non-homologous end joining
OPC	oligodendrocyte precursor cell
ORF	open reading frame
PAM	protospacer adjacent motif
PAMP	pathogen-associated molecular patterns
PD	Parkinson’s disease
PNS	peripheral nervous system
PRR	pattern recognition receptors
RGMa	repulsive guidance molecule A
RNP	ribonucleoprotein complex
sgRNA	single guide RNA
TALEN	transcription activator-like effector nuclease
TLR	Toll-like receptor
VLP	virus-like particle
ZNF	zinc finger nuclease
